# Comparison of immunoglobulin Y antibody production in new and spent laying hens

**DOI:** 10.14202/vetworld.2024.2177-2184

**Published:** 2024-09-28

**Authors:** Pornphimon Metheenukul, Win Surachetpong, Noppadol Prasertsincharoen, Peera Arreesrisom, Naris Thengchaisri

**Affiliations:** 1 Department of Veterinary Technology, Faculty of Veterinary Technology, Kasetsart University, Bangkok, Thailand; 2 Department of Veterinary Microbiology and Immunology, Faculty of Veterinary Medicine, Kasetsart University, Bangkok, Thailand; 3 Department of Companion Animal Clinical Sciences, Faculty of Veterinary Medicine, Kasetsart University, Bangkok, Thailand

**Keywords:** antibody production, diagnosis, immunoglobulin Y antibody, immunological assays, spent laying hens

## Abstract

**Background and Aim:**

Immunoglobulin (Ig)Y, a specific type of Ig found in chicken eggs, has potential use in the diagnosis of human and animal diseases. This study assessed the feasibility of using spent laying hens to produce IgY. In addition, the effects of antigen injection on egg and antibody production in new and spent laying hens were compared.

**Materials and Methods:**

Hens were intramuscularly injected with three booster shots of antigens. IgY was extracted from egg yolks using polyethylene glycol 6000 precipitation followed by dialysis.

**Results:**

Spent laying hens (83 weeks) consistently showed lower egg production than new laying hens (27 weeks) throughout the study. Post-immunization, a further decline in egg production was observed in spent laying hens, and egg production stopped after the second antigen injection. Eggs from spent laying hens were less dense than eggs from new hens. Despite lower IgY levels in eggs from spent laying hens, the heavy-to-light chain ratio remained consistent in both groups. Notably, IgY from spent and new laying hens demonstrated effective hemagglutination against cat erythrocytes in the A blood group.

**Conclusions:**

This study demonstrated the potential of using spent laying hens to produce IgY, with significant implications for future research, immunotherapy, and diagnostic applications, despite the observed reduction in egg production compared with new laying hens.

## Introduction

Antibodies serve as important tools with numerous applications in life science and translational research in medicine. Polyclonal and monoclonal antibodies, each with their own advantages and limitations, are important to research [1]. Among them, immunoglobulin (Ig) Y, a polyclonal antibody found predominantly in egg yolk, has significance in diagnostic applications. Although primarily identified in avian species, IgY shares structural similarities with mammalian IgG antibodies [2]. In avian biology, IgY plays an important role in passive immunity during the early life stages of birds, protecting embryos through efficient transfer to egg yolks [3]. IgY technology has gained considerable attention over recent decades, particularly for its sensitive detection of biomolecules and its ability to be applied to human and animal diagnoses [4].

Remarkably, hens have the potential to produce antibodies, making them a practical source of antibodies. High antibody production yields in hen eggs fulfill not only ethical considerations, such as the principle of the 3Rs concept (reduction, replacement, and refinement) but also offer economic advantages over conventional animal antibody production [2, 4–6]. Notably, the noninvasive isolation of IgY antibodies from egg yolks distinguished hen-based production methods from other animal systems, mitigating ethical concerns associated with conventional antibody retrieval methods through blood collection. Moreover, hens have lower maintenance costs than other commonly used animal models, including mice and rabbits [7]. Despite these advantages, IgY yields from hens are comparable to those from larger animals, such as goats and sheep, with an estimated production of 18–25 g of IgY per year per hen [3, 8]. Furthermore, specific IgY antibodies constitute a significant portion, ranging from 1% to 10% of the total antibodies produced by hens [3]. However, various factors, including antigen dosage, immunogenicity, and molecular weight, influence antibody production in each hen [2, 3].

IgY has been utilized in various applications to improve both human and veterinary health, including immunodiagnostics [3, 4, 9–11], immunotherapy [12, 13], passive immunization [14, 15], neutralization of toxins from venomous animals [16, 17] and bacteria [8, 18–20], and production of functional foods [21, 22]. Despite its potential, the expansion of the IgY product range has been relatively slow because of infrastructure costs, the pricing of raw antibodies or ready-to-use products, and limited market demand [23]. Nevertheless, IgY production remains cost effective, particularly when extracted from egg yolks [24]. To optimize IgY production costs and increase efficiency in the use of hens for antibody production, a study should be conducted comparing the use of spent and new laying hens. A previous study by Marzec *et al*. [25] indicated that both the functional and physicochemical properties of fresh eggs remain unaffected by hen age, making spent laying hens an interesting option for IgY production.

This study aimed to determine the feasibility of using spent laying hens to produce polyclonal IgY. Furthermore, by comparing outcomes between spent and new laying hens, the study assessed the impact of antigen injection on egg and IgY production and the application of IgY for hemagglutination potential using cat red blood cells (RBCs).

## Materials and Methods

### Ethical approval

This study was approved by the Institutional Animal Care and Use Committee of Kasetsart University (protocol number ACKU64-VET-018) and was in accordance with the principles of the 3Rs concept.

### Study period and location

The study was conducted from July 2021 to December 2021. Eight hens were enrolled in the present study at the Animal Facility of the Faculty of Veterinary Medicine, Kasetsart University, Bang Khen, Bangkok, Thailand.

### Ghost cell preparation

Blood samples from two cats (A and B blood groups) and two dogs (dog erythrocyte antigen [DEA 1.1]), each containing 2 mL, were centrifuged at 4000× *g* for 3 min to separate plasma, followed by removal of the buffy coat. The packed red cells were washed 3 times with phosphate-buffered saline (PBS) at pH 7.4. Subsequently, the washed red cells were lysed in a hypotonic buffer. The lysed red cells were centrifuged at 4000× *g* for 10 min and washed 3 times with hypotonic buffer (containing 7 mM NaCl and 5 mM Tris-HCl at pH 7.4) to eliminate any remaining hemoglobin. The ghost cells were then aliquoted and stored at −20°C.

### Immunization of chickens and egg collection

The hens (*Gallus gallus domesticus*) in this study were divided into two groups based on age: Spent laying hens (age 83 weeks) and new laying hens (age 27 weeks). Each group was inoculated with RBCs prepared from feline and canine blood samples. The group of spent laying hens comprised four chickens: One as a control, one injected with ghost cells from dog blood type DEA 1.1, one injected with ghost cells from cat blood type A, and another injected with ghost cells from cat blood type B. The group of new laying hens consisted of four chickens: One injected with 1 mg/mL synthetic peptide, one injected with 2 mg/mL synthetic peptide, one injected with ghost cells from type A cat blood, and one injected with ghost cells from type B cat blood.

For immunization, a concentration of 0.3 mg/mL of either A-positive or B-positive feline ghost cells or DEA1.1-positive canine ghost cells was used, then mixed with an equal volume of Freund’s complete adjuvant (Sigma, St Louis, MO, USA) for the initial injection. Subsequent boosters were applied to equal volumes of Freund’s incomplete adjuvant (Sigma). These inoculations were administered at 1-week intervals through intramuscular injection into the musculus pectorals of the chicken, with a final volume of 1.0 mL evenly distributed across the two injection sites. Throughout the experimental period, there was no evidence of pain, discomfort, palpable reaction edema, or tissue damage at the inoculation sites, as well as mortality. Eggs were collected daily before and after immunization and stored at 4°C. The hens maintained their overall well-being without any signs of developmental anomalies during the immunization process.

### Extraction of total IgY

Total IgY was extracted from egg yolk by precipitation with polyethylene glycol 6000 (PEG 6000) following the method by Hadi *et al*. [26]. This method involves two key steps: Lipid removal and precipitation of total IgY from the supernatant obtained during the initial step. The eggshells were carefully broken, and yolk was isolated, after which egg white and albumen. The yolk was then rolled on filter paper to eliminate the remaining egg white. A pipette tip was used to incise the yolk skin, and the yolk was transferred to a 50 mL tube with its volume recorded. The yolk was then mixed with two volumes of PBS and PEG 6000 to obtain a final concentration of 3.5% PEG 6000. The mixture was vortexed for 10 min and then centrifuged at 13,000× *g* at 4°C for 20 min. The supernatant was carefully collected through a folded filter into a new tube, and 8.5% PEG 6000 was added. Samples were vortexed and pellets were harvested by centrifugation under the same conditions as previously described. The supernatant was discarded, and the pellet was dissolved in 1 mL of PBS, with additional PBS added to reach a final volume of 10 mL. The solution was thoroughly mixed with 12% PEG 6000 (w/v, 1.2 g) and vortexed. The IgY extract was subsequently dialyzed against 0.1% NaCl for 3 h in PBS at 4°C and stored at −20°C.

### Protein quantitation and characterization of IgY by sodium dodecyl sulfate-polyacrylamide gel electrophoresis (SDS-PAGE)

The protein concentrations (mg/mL) in the samples were determined photometrically at 280 nm (diluted 1:10 with PBS) using a UV1800 Spectrophotometer (Shimazu, Japan) and calculated using the Lambert–Beer law. Following isolation and purification, IgY was separated by SDS-PAGE using a Protean II electrophoresis system (Bio-Rad, USA) and a discontinuous buffer system [27]. The IgY samples were subjected to 10% SDS-PAGE under reducing conditions, and electrophoresis was performed at 120 V for 80 min. Visualization of the proteins was achieved through staining with Coomassie Blue.

### Hemagglutination

The hemagglutination test was conducted in microtiter plates. Initially, 0.1 mL of IgY (anti DEA1.1) and concentrated IgY (anti-A or anti-B) were serially diluted in two-fold steps with PBS at pH 7.4. Subsequently, 0.1 mL of 2% feline RBC A- or B-positive samples was added to the diluted antibody. The mixture was incubated at room temperature (25°C) for 1 h and then kept at 4°C overnight.

### Statistical analysis

Data represent the means ± standard deviation of three independent trials calculated using Prism 6.0 (GraphPad Software, Boston, MA). Egg production was compared between spent and new laying hens using repeated measures one-way analysis of variance and *post hoc* Tukey tests. The comparison of egg weights (whole eggs, egg yolks, and egg whites) and IgY production between spent and new laying hens was conducted using Student’s t-test. The significance level was set at p < 0.05.

## Results

**Figure-1:**
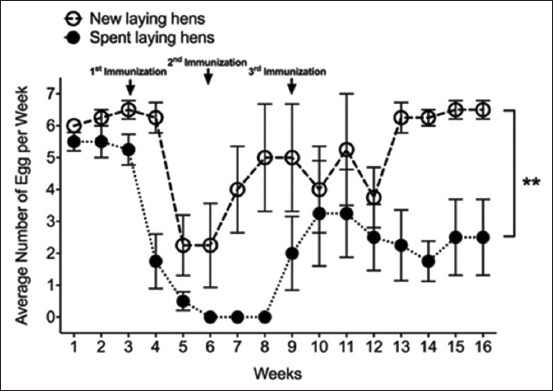
Comparison of laying capacity between spent laying hens (83 weeks old, n = 4) and new laying hens (27 weeks old, n = 4) after three consecutive immunization periods during a 16-week experiment. Data were analyzed using analysis of variance and *post hoc* Tukey test (p < 0.05).

**Figure-2:**
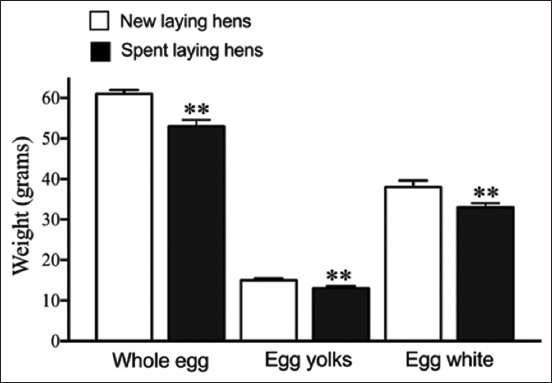
Comparison of the average weights of whole eggs, egg yolks, and egg whites between new and spent laying hens. Data were analyzed using analysis of variance and post hoc Tukey test (p < 0.01).

Spent laying hens and new laying hens received intramuscular injections of antigen and adjuvant 1 week apart, with daily egg collection and storage at 4°C for further study. Spent laying hens and new laying hens did not exhibit abnormalities, deaths, pain, discomfort, edema, or tissue damage at the injection sites. The data obtained from the animal experiments are presented in the Table-1. Egg-laying performance of hens immunized with various antigens was monitored daily for 16 weeks. The following antigen administration in spent and new laying hens, there was a decrease in egg production. Egg production stopped after the second antigen injection. Although egg production resumed in spent laying hens, it remained consistently lower than that of new laying hens throughout the experiment (Figure-1). However, the overall egg quantity of new laying hens was significantly greater than that of spent laying hens. (5.13 ± 1.31 vs. 2.39 ± 1.17 eggs per week, p < 0.01; Table-1). The difference between spent and new laying hens in the production of antibodies from egg yolk has been demonstrated. Eggs from both spent and new laying hens were collected daily before and after immunization and stored at 4°C. All eggs were weighed, followed by separation and weighing of yolks and egg whites. Whole eggs from spent laying hens weigh an average of 59.34 ± 2.00 g, with 33.54 ± 1.63 g consisting of egg white and 13.10 ± 1.18 g consisting of yolk. In comparison, eggs from new laying hens are slightly heavier, averaging 61.38 ± 2.03 g, with 38.63 ± 1.73 g of egg white and 15.05 ± 1.86 g of yolk. From statistical analysis, it was found that the average weight of whole eggs, egg yolks, and egg whites in spent laying hens was significantly lower than that in new laying hens (Figure-2) p < 0.05.

**Table-1:** General characteristics of new and spent laying hens for antibody production.

Parameters	New laying hens	Spent laying hens
Number of animals	4	4
Age	27 weeks	83 weeks
Number of weeks	16 weeks	16 weeks
Total number of eggs	328	153
Average number of eggs per hen	82.00 ± 21.02	38.25 ± 18.75**
Average number of eggs per hen per week	5.13 ± 1.31	2.39 ± 1.17**

** p < 0.01 versus new laying hens

**Figure-3:**
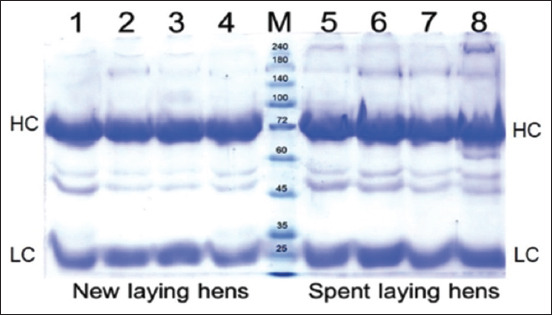
SDS-PAGE profiles of individual IgY preparations from different eggs under reducing conditions. The two IgY chains: HC at 69 kDa and LC at 24 kDa, were separated using 10% SDS-PAGE gels. Lanes 1–4 show IgY purified from new laying hens, where “M” denotes an unstained protein ladder. Lanes 5–8 show purified IgY from spent laying hens, where HC indicates heavy chains and LC indicates light chains. The data represent the experimental results. The results of the other experiments were performed in the same manner. SDS-PAGE= Sodium dodecyl sulfate-polyacrylamide gel electrophoresis, HC= Heavy chain, LC= Light chain, IgY= Immunoglobulin Y.

The purity and yield at each stage of egg yolk purification are summarized in Table-2. The purified protein from spent laying hens was significantly higher than that from new laying hens (12.98 ± 1.96 mg/mL vs. 6.55±0.58 mg/mL, p < 0.01). After precipitation with 3.5% PEG, distinct bands with higher PEG contents were effectively eluted. The band intensity gradually decreased as the PEG concentration increased to 8.5%. Some bands vanished as the PEG concentration reached 12%. PEG and minor impurities were removed using dialysis, while SDS-PAGE analysis was employed to confirm the content and purity. Additional bands between the heavy and light chains of IgY were observed, suggesting minor impurities with molecular weights ranging from 69 to 24 kDa (Figure-3). Notably, the presence of the heavy chain at 69 kDa and the light chain at 24 kDa was confirmed by SDS-PAGE. Other bands between the heavy and light chains of IgY were identified as minor impurities. This is an example from spent and new laying hens. All eggs from the remaining group of hens produced the same results. Immunization with various antigens to produce antibodies from egg yolk may be a difference between spent and new laying hens.

**Table-2:** Purity and yield measurements at each purification stage from egg yolk.

Subjects	Protein concentration (mg/mL)			

	3.5% PEG	8.5% PEG	12% PEG	After dialysis
New laying hens	10.18 ± 0.53	6.29 ± 0.24	0.92 ± 0.07	6.55 ± 0.58
Spent laying hens	11.86 ± 1.07	6.24 ± 0.75	1.00 ± 0.06	12.98 ± 1.96**

PEG: Polyethylene glycol.

** p < 0.01 versus new laying hens

**Figure-4:**
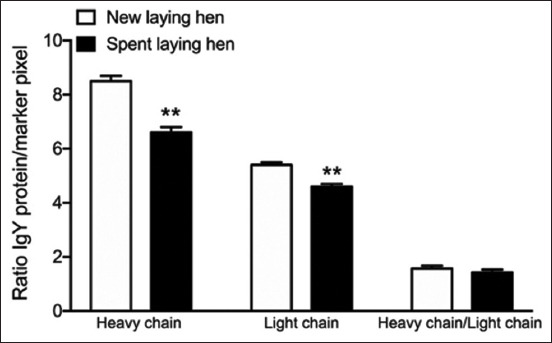
The ratio of immunoglobulin Y protein pixel intensity to marker pixel intensity in eggs from both new laying hens (n = 4) and spent laying hens (n = 4). **p < 0.01 versus new laying hens.

**Figure-5:**
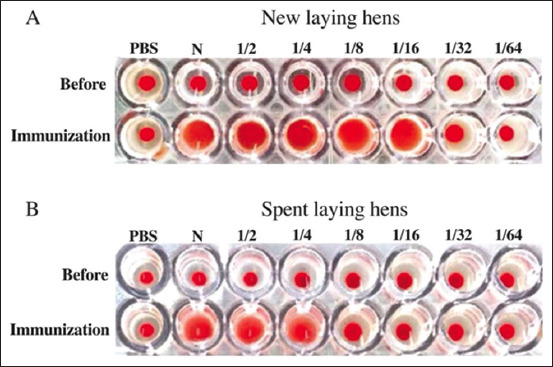
Hemagglutination assay demonstrating IgY antibody reactions against 2% A-positive cat erythrocytes. Panel A presents antibodies derived from new laying hens and panel B presents antibodies derived from spent laying hens.

Heavy and light chains were analyzed during gel protein separation in pixel format, with the pixel density representing the concentration. The heavy chain of new laying hens was 8.50 ± 0.20 pixels/unit and the light chain was 5.40 ± 0.10 pixels/unit, with the ratio of heavy chain to light chain being 1.57 ± 0.10 pixels/unit. The heavy chain of spent laying hens was 6.60 ± 0.20 pixels/unit, and the light chain was 4.60 ± 0.10 pixels/unit, with the ratio of heavy chain to light chain being 1.43±0.10 pixels/unit. The concentrations of IgY heavy and light chains in spent laying hens were significantly lower than those in newly introduced laying hens. However, the ratio of heavy chain to light chain was not significantly different between spent and newly introduced laying hens (Figure-4). In addition, hemagglutination against ghost RBCs from cats with the A blood group was achieved using IgY from both spent laying hens and newly introduced laying hens (Figure-5). Specifically, hemagglutination of cat samples and polyclonal antibody against the A blood group with diluted IgY from new laying hens was observed at a titer of 16, whereas hemagglutination of cat samples and polyclonal antibody against the A blood group with diluted IgY from spent laying hens was observed at a titer of 4, suggesting that the new laying hens had higher specific antibody levels than the spent laying hens.

## Discussion

Serum IgY in hens is transferred to egg yolk to provide natural immunity to chicks. Hen antibodies are acquired by chicks through the latent stage and play an important role in their immune system, protecting them from various infectious diseases [2, 28]. The primary aim of this study was to evaluate the feasibility of using spent laying hens to produce polyclonal IgY. In addition, we investigated the impact of antigen injection on egg production and antibody generation in spent and new-laying hens.

Antibody production in spent and new laying hens was compared. All hens were healthy and did not exhibit any abnormalities. Monitoring egg production revealed that eggs from some hens had decreased albumen and egg yolk content after antigen injection. These results are consistent with those of previous studies on eggs from spent laying hens [3, 11, 25, 29]. However, eggs from spent laying hens (69–70 weeks of age) exhibited similar quality as eggs from younger hens (45 to 46 and 55 to 56 weeks of age) [25]. The relative ease of IgY purification renders it a low-cost and attractive antibody for research and diagnosis [3, 29]. Purification of IgY from egg yolk was dependent on combining the PEG and dialysis methods although some contaminating proteins were present in the preparations. The extraction procedure utilized in this study separated egg yolk into two phases: One consisting of yolk solids and fatty substances and a second watery phase containing IgY and other proteins. Crude IgY extracts and purified IgY products were obtained by sequential PEG precipitation of egg yolks. This procedure removed lipoproteins, and IgY antibody-lipoprotein complexes were the major products, although a small amount of lipoprotein was present [30]. The results indicate that the combination of PEG and dialysis is essential for improving the purity of the resulting product.

The total IgY content has been reported to increase with the age of the hen, from approximately 40 mg/egg to 80 mg/egg [9]. In our analysis, the content of IgY protein isolated from eggs collected from new laying hens was higher than that isolated from spent laying hens. However, the ratio of heavy and light IgY chains was similar between the two groups. This difference may be due to the larger molecular mass of the IgY heavy chain due to more heavy-chain constant domains and carbohydrate modifications. Furthermore, the less flexible hinge region of IgY compared with mammalian IgG suggests that IgY is a more hydrophobic molecule, which may affect its mobility [31].

Egg-derived IgY is the primary Ig in avian serum produced by B cells in response to antigens, similar to mammalian IgG, but with distinct molecular characteristics. Structurally, IgY features larger heavy chains (approximately 65,100 amino acid units) and slightly smaller light chains (about 18,700 amino acid units) than IgG, resulting in a molecular weight of approximately 180 kDa. IgY lacks a hinge region, making it inflexible, yet it plays a critical role in the avian immune response against pathogens. Notably, IgY has demonstrated effectiveness in various scientific, diagnostic, prophylactic, and therapeutic applications, including in immunological assays, due to its ability to recognize specific antigens, low cross-reactivity with mammalian IgG, and inactivation of the mammalian complement system [3, 8, 32]. Hemagglutination assays demonstrated that IgY antibodies from immunized spent-laying and new-laying hens were reactive against cat erythrocytes in the A blood group. These findings are consistent with those of other studies describing the successful application of IgY for scientific, diagnostic, prophylactic, and therapeutic purposes, as well as immunochemical reagents [2, 3]. Indeed, IgY is an excellent type of antibody that could be used in various immunological assays because it reacts to different rheumatoid factors and human anti-mouse IgG antibodies. Another benefit of IgY is its ability to inactivate the mammalian complement system and not bind to Fc receptors [3]. Furthermore, IgY shows poor cross-reactivity to mammalian IgG because of differences in the heavy-chain structure.

The production of IgY in hens is straightforward and offers several advantages, including the absence of a blood collection process, the need for only eggs following immunization, and low amounts of antigen required to obtain high and long-lasting IgY titers in the yolk of immunized hen eggs [3]. Therefore, the production of polyclonal antibodies through chicken immunization makes IgY an excellent alternative for the production of large and high-quality antibodies through simple production methods that are not invasive [3]. Moreover, the binding of different epitopes and/or antigens by extractable polyclonal antibodies allows the application of IgY under different immunological conditions. Importantly, this feature makes polyclonal IgY antibodies well-suited for many diagnostic applications [1, 3]. Although monoclonal antibodies offer high specificity, they are costly and labor intensive, require specialized facilities, and the extensive production of monoclonal antibodies often involves complex optimization and purification processes. In contrast, using a sustainable resource and avoiding invasive procedures are alternative procedures for resource-limited antibody production facilities.

**Figure-6:**
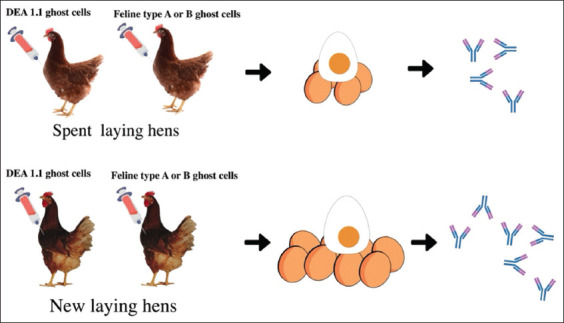
A drawing illustrates the potential of spent laying hens as a promising source of antibody production. Despite their significantly lower egg production than new laying hens, hens have the potential to be an alternative source of sustainable antibody production.

Traditional approaches to developing antiviral, antibacterial, and antiparasitic therapeutics are often hindered by slow production, high costs, and the emergence of pathogen resistance [3, 8, 32]. Chicken IgY has been widely explored as a clinical anti-infective material for prophylaxis, preventive medicine, and infectious disease therapy. IgY neutralizes viruses through mechanisms such as blocking viral attachment, preventing membrane fusion, promoting viral detachment, interfering with free virions, and inducing viral aggregation for immobilization. Although chickens are primarily used for IgY production due to their efficient and cost-effective egg-laying, other avian species, such as geese, ducks, ostriches, and quails have also been successfully employed to generate IgY antibodies effective against pathogens, such as dengue, West Nile, Zika, Hantavirus, and *Helicobacter pylori* in various experimental models [3, 8, 32]. Non-systemically administered IgY antibodies are safe and effective drugs [29]. Moreover, passive immunization with avian antibodies could represent an effective alternative therapy because IgY antibodies can be obtained relatively simply, cost-efficiently, and produced on a large scale [2, 24]. The present study demonstrated the viability of spent laying hens as a source for antibody production, despite lower egg production in spent laying hens than in new laying hens (Figure-6).

The limitations of the study include the lack of investigation into specific factors contributing to the cessation of egg production in spent laying hens following antigen administration. Immunization-linked stress may be partially responsible for decreased laying rates observed in both spent and new laying hens. Moreover, this study did not evaluate the consistent quality and quantity of antibody production. Establishing robust criteria for evaluating antibody quality and demonstrating factors such as specificity, functionality, stability, and reproducibility is crucial for a comprehensive assessment beyond purity and yield measurements.

## Conclusion

The present study demonstrated the potential of using spent laying hens to produce polyclonal IgY antibodies, demonstrating their ability to produce substantial quantities of high-quality antibodies through a straightforward and practical process. Chicken IgY antibodies purified from the egg yolks of immunized hens exhibit reactivity to various antigens, supporting the use of IgY as an alternative to more invasive and costly methods. Despite the challenges of reduced egg production observed in spent laying hens compared with new laying hens, the ease and cost-effectiveness of IgY purification, along with its low cross-reactivity with mammalian IgG, make it an attractive option for research, diagnosis, and immunotherapy. These findings underscore the importance of immunizing chickens before they begin egg production, as stress induced by handling during the laying phase can negatively impact egg output. Moreover, IgY production offers a viable alternative for laboratories with limited technology, allowing them to raise chickens to produce antibodies for diagnostic testing or other biomedical purposes instead of relying on monoclonal antibodies.

## Authors’ Contributions

PM, WS, and NT: Conceptualization, data analysis, and writing-review and editing. PM, NP, PA, and NT: Conducted experiments. PM, NP, and PA: Methodology. PM and WS: Writing-original draft preparation. All authors have read and approved the final version of the manuscript.
